# Unveiling the 3D Morphology of Epitaxial GaAs/AlGaAs Quantum Dots

**DOI:** 10.1021/acs.nanolett.4c02182

**Published:** 2024-07-25

**Authors:** Yiteng Zhang, Lukas Grünewald, Xin Cao, Doaa Abdelbarey, Xian Zheng, Eddy Patrick Rugeramigabo, Johan Verbeeck, Michael Zopf, Fei Ding

**Affiliations:** † Institut für Festkörperphysik, Leibniz Universität Hannover, Appelstraße 2, 30167 Hannover, Germany; ‡ EMAT, University of Antwerp, Groenenborgerlaan 171, B-2020 Antwerp, Belgium; § Laboratorium für Nano- und Quantenengineering, Leibniz Universität Hannover, Schneiderberg 39, 30167 Hannover, Germany

**Keywords:** GaAs/AlGaAs, semiconductor quantum dots, 3D morphology, HAADF-STEM, selective chemical etching, AFM

## Abstract

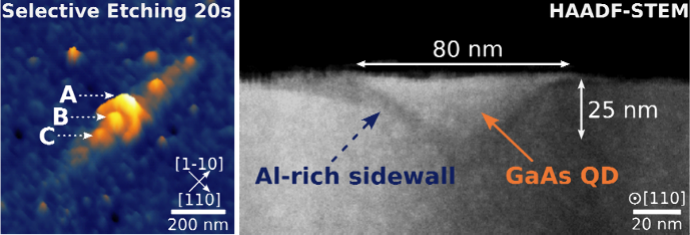

Strain-free GaAs/AlGaAs semiconductor quantum dots (QDs) grown by droplet etching and nanohole infilling (DENI) are highly promising candidates for the on-demand generation of indistinguishable and entangled photon sources. The spectroscopic fingerprint and quantum optical properties of QDs are significantly influenced by their morphology. The effects of nanohole geometry and infilled material on the exciton binding energies and fine structure splitting are well-understood. However, a comprehensive understanding of GaAs/AlGaAs QD morphology remains elusive. To address this, we employ high-resolution scanning transmission electron microscopy (STEM) and reverse engineering through selective chemical etching and atomic force microscopy (AFM). Cross-sectional STEM of uncapped QDs reveals an inverted conical nanohole with Al-rich sidewalls and defect-free interfaces. Subsequent selective chemical etching and AFM measurements further reveal asymmetries in element distribution. This study enhances the understanding of DENI QD morphology and provides a fundamental three-dimensional structural model for simulating and optimizing their optoelectronic properties.

Semiconductor quantum dots (QDs) embedded within a single-crystalline host matrix represent exceptional sources of nonclassical photons, playing a pivotal role in quantum technologies.^1−3^ The GaAs/AlGaAs QDs produced through droplet etching and nanohole infilling (DENI) are highly attractive due to their anticipated minimal crystal defects^4^ and outstanding optical properties, excelling in key aspects such as pure,^5^ bright,^6,7^ and indistinguishable^8,9^ single-photon emission with line widths close to Fourier limit,^10−12^ as well as strong multiphoton entanglement.^13,14^ After enhancing their optical properties through postgrowth techniques, such as electrical fields,^6,15^ strain fields,^13,16,17^ and optical cavities,^18^ they can be utilized in a variety of quantum photonic systems, including entanglement swapping^19,20^ or quantum key distribution.^21^ It is important to note that understanding and controlling the morphology of DENI QDs, in particular, their symmetry and material composition, is essential to achieve the desired optical properties. For example, QDs obtained by filling highly symmetric nanoholes show nearly vanishing fine structure splitting (FSS) and thus improve the fidelity of polarization entanglement.^17,22^ Intrinsic anisotropic strain fields and in-plane anisotropy of the confinement potential influence the splitting and mixing of heavy and light holes.^23^ Smaller QDs tend to weaken the hyperfine interaction caused by the distance between electrons and nuclear spins and also show a significant inhibitory effect on spin–orbit coupling, thereby extending the spin coherence time.^24,25^ In addition, reducing the compositional intermixing of AlGaAs and GaAs in the QDs will further extend the spin coherence time.^25,26^ The energy of the confined exciton in DENI QD is mainly determined by the confinement in the growth direction and the infilling amount of GaAs. As a result, different filling amounts realize a wide distribution of exciton emission wavelengths.^27−29^ Meanwhile, the weak confinement due to large lateral dimensions, which often exceed the free exciton Bohr radius in GaAs, shortens the exciton radiation lifetime.^30^ Therefore, a comprehensive understanding of the three-dimensional (3D) morphology of the DENI-based QD system is essential for optimizing the optical properties intrinsically.

Various techniques are employed to study the morphology of QDs, including atomic force microscopy (AFM),^28,31^ cross-sectional scanning transmission electron microscopy (STEM),^24,32,33^ and chemical etching.^31,34,35^ AFM provides valuable information about the depth, symmetry, density, and structure details of droplet-etched nanoholes, such as the facet formation.^36^ However, the morphology investigation of filled nanoholes has received little attention, a critical aspect that directly impacts the comprehension of their optical properties. TEM and STM are effective tools for analyzing crystal strain defects, material composition, and interfacial properties in both strained InGaAs^33^ or unstrained droplet epitaxy GaAs^37^ QDs, respectively. Nonetheless, achieving precise nanoscale localization can be challenging regarding the lower density of DENI nanostructures; thus, only a few studies have been conducted on the morphology of DENI QDs.^24^ Selective wet etching can complement and thus compensate limitation of AFM and TEM, which can only perform two-dimensional characterization. Etch rate increases around the defect sites, such as dislocations or stacking faults, and causes etch pit formation.^38−40^ In combination with AFM, the 3D alloy composition and distribution of In(Ga)As^31^ and SiGe^41^ QDs has been successfully obtained, while the composition distributions and conductance distributions of GeSi quantum rings^42^ have been detected. This method provides a fast, intuitive, and controllable way to study the QD morphology. It is worth noting that although each method has its limitations, it is promising to achieve a comprehensive understanding of the 3D morphology of GaAs/AlGaAs by using multiple and complementary methods.

Here, we comprehensively demonstrate the first full-scale 3D morphology of DENI QDs. To do this, we use high-resolution STEM to investigate the cross-section morphology, revealing asymmetric sidewalls and Al-rich regions in the nanohole. The lack of crystal defects and strain suggests that the QDs have coherently crystallized within the nanohole. Selective chemical etching combined with AFM is then employed to further analyze the composition, structure, and distribution in size and symmetry. Our work lays the foundation for morphology analysis of DENI QDs and provides a precise physical model for future studies.

The GaAs/AlGaAs QDs analyzed in this study are grown using solid-source molecular beam epitaxy with *in situ* DENI^43−44,45^ method (Supporting Information). The formation of QDs involves a significant mixing of involved atoms at the interface to the nanohole, leading to an inhomogeneous element distribution. These “uncapped QDs”, which are not contaminated by Al from the capping layer and are easily localized by STEM, are the optimal choice to study the DENI QD morphology using selective chemical etching. To understand the morphology of the nanohole-to-QD structure, we grow a series of uncapped QDs with different filling amounts (Figure 1).

**Figure 1:**
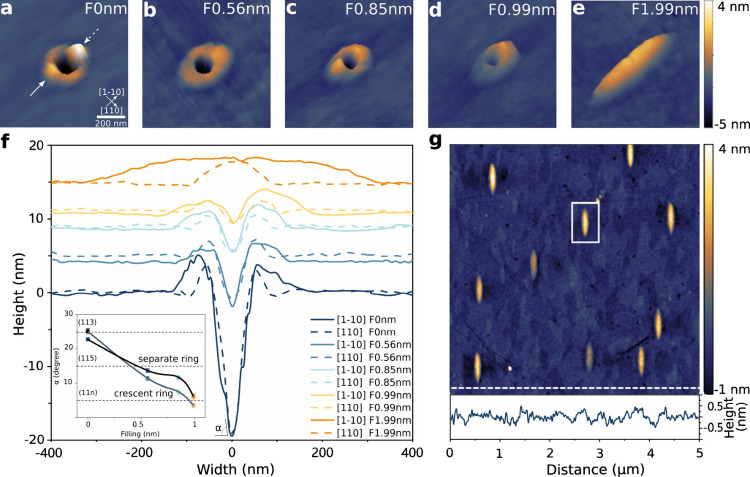
Morphology of GaAs QDs with varying infilling amounts. Panels (a) to (e) illustrate the AFM tilted view (30°) morphology change from nanohole to filled QDs, corresponding to filling amounts of 0, 0.56, 0.85, 0.99, and 1.99 nm, respectively. The nominal amount of GaAs filling is denoted as F*X* nm. The crescent-shaped part of the nanoring faces random orientations, either [1–10] or the opposite. The solid and dashed arrows in (a) represent the crescent and separate parts of the nanoring, respectively. (f) compares the QD line profile along the [1–10] and [110] orientations. As the amount of infillings increases, the outer diameter of the nanoring increases from 240 to 265, 285, 340, and 700 nm along the [1–10] orientation. The inset illustrates the correlation between the main facet of the nanohole to (001) crystal plane angle α and the amount of filling. A larger facet index (11n) results in a faster growth/etch rate. The F1.99 nm sample has a root-mean-square (RMS) roughness of 0.2 nm, and its surface morphology is shown in (g). The roughness curve along the [110] orientation (dashed white line at the bottom of (g)) indicates the layer-by-layer growth of the sample over 5 μm. (e) is a magnified view of the QD in a white box in (g).

We first obtain nanoholes etched by Al droplets as shown in Figure 1a. The surface of the nanohole is surrounded by an asymmetric nanoring consisting of a larger crescent-shaped part and a separate but higher part^28,36^ (cf. solid and dashed arrows in Figure 1a, respectively). This asymmetry is attributed to the ripening direction of Al droplet.^46^ The nanohole openings are subcircular with a slightly shorter width of approximately 68 nm in the [110] orientation compared to 83 nm in the [1–10] orientation. As shown in Figure 1f for the unfilled nanohole (denoted as F0 nm), the contour lines in the two orthogonal directions indicate that the as-etched nanoholes are in the shape of an inverted cone, with an average depth of 22 nm in both orientations. The nanoring shows a rough surface, and the inner wall of the inverted cone exhibits a multidirectional faceted morphology.^36^

After the droplet etching process, we introduce different amounts of GaAs material, leading to changes in the morphology from Figure 1b to 1e. The size of the nanohole opening gradually decreases and the volume of the rings increases along the [1–10] orientation. When overfilled to 1.99 nm (Figure 1e), the nanohole completely disappears and merges with the nanoring into an elliptical bump due to the tendency to reduce the surface energy (Figure 1f). The behavior is attributed to the differences in surface area demonstrated by the increased difference in facet angles^36,47^ (inset of Figure 1f). Due to the preferential migration in the [1–10] orientation during GaAs epitaxy, the growth results in an elliptical bump within an average length of approximately 700 nm (Figure 1g), an average height of 3 nm, and a pitted surface^28,48^ (Figure S 1d). Therefore, a typical GaAs QD is produced, consisting of an elliptical bump in the upper part and an inverted cone in the lower part.

We randomly select an elliptical bump QD for TEM characterization (Supporting Information). The nanostructure and chemical composition of the QD are analyzed by TEM of a cross-sectionl sample as shown in Figure 2. The contour of the QD region is visible in *Z*-contrast high-angle annular dark-field (HAADF) STEM imaging, where the local image intensity is roughly proportional to the atomic number *Z*^1.7^(49,50) (Figure 2a). The QD has an inverted triangular shape with a width of approximately 80 nm at the surface and grows about 25 nm into the Al_0.23_Ga_0.77_As barrier. These dimensions are similar to the nanohole sizes (Figure S1e), indicating that the cross-section is at the center of the QD. Chemical analysis using energy-dispersive X-ray spectroscopy (EDS) reveals an increased Ga signal and Al depletion in the QD region (Figure 2b), as expected for GaAs relative to Al_0.23_Ga_0.77_As. The observation mentioned above is also evident in the summed-up EDS spectra (Figure 2c) of the QD and Al_0.23_Ga_0.77_As regions. The Ga peak intensities are higher in the triangular QD region (cf. solid arrows in Figure 2c), whereas the Al signal is reduced (dotted arrow in Figure 2c).

**Figure 2:**
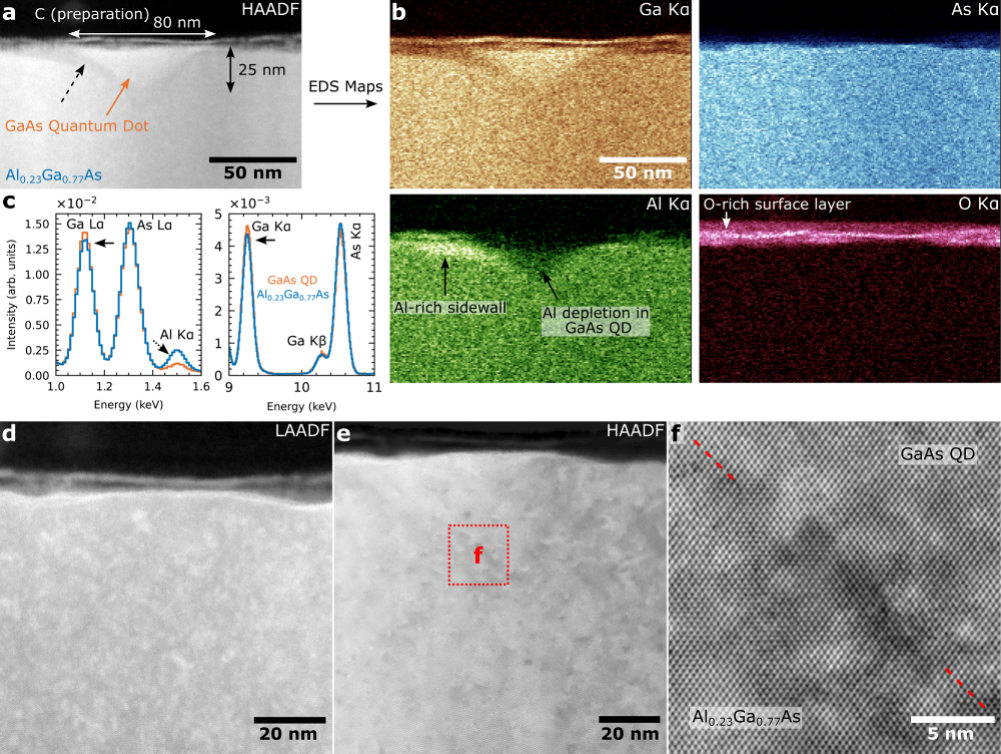
Microstructural characterization of the QD core by TEM. (a) Overview cross-section HAADF-STEM image of a 25 nm thin GaAs QD at the surface of an Al_0.23_Ga_0.77_As matrix. The black dashed arrow represents an area of enhanced Al signals, and the yellow arrows represent the QD “core” region. The TEM sample thickness is about 75 nm. (b) Elemental maps of selected elements from the region shown in (a). The GaAs QD shows Al depletion, slight Ga enrichment, and no change in As signals. Oxidation of the sample leads to an oxidized surface layer. Al-rich asymmetric sidewalls on the left and right sides can be seen in the Al map. (c) Qualitative comparison of summed-up and normalized EDS spectra from the GaAs QD and Al_0.23_Ga_0.77_As regions. Note the depletion (enrichment) of the Al (Ga) signal in the QD region relative to the matrix, marked with dotted (solid) arrows. The EDS spectra intensities were normalized to the total number of X-ray counts in each spectrum for easier comparison. (d) LAADF-STEM image of the QD showing no visible intensity changes at the QD-matrix interface, indicating no/low strain or other crystalline defects. (e, f) Overview and higher-magnification HAADF-STEM image of the QD-matrix interface. The dashed line roughly marks the expected position of the interface.

An increase in the Al signal is visible on the QD sidewall (Figure 2b), which also explains the reduced HAADF-STEM intensity in Figure 2a (dashed black arrow). The As signal is constant in this field of view. The latter observation may indicate that the sidewall is composed of an AlAs layer, as the nominal As concentration (50 at%) is the same for Al_0.23_Ga_0.77_As, GaAs, and AlAs (Figure 2b). The effective atomic number *Z*_eff_(51,52) of AlAs (*Z*_eff_ = 24.1) is noticeably lower than that of the GaAs QD (*Z*_eff_ = 32.0) and Al_0.23_Ga_0.77_As (*Z*_eff_ = 30.3), leading to a reduced HAADF-STEM intensity. Additionally, we find that the Al-rich sidewall thickness varies, with a side region of approximately 5–8 nm and increasing to 15 nm at the bottom of the inverted cone (Figure S3b). The asymmetric Al signal around the nanohole observed by STEM-EDS (Figure 2b) is in agreement with the asymmetric sidewalls observed by AFM (Figure 1a). The Ga concentration is maximum at the top of the QD “core” region and gradually decreases to a minimum at the bottom, while the Al concentration exhibits the opposite trend, reaching its highest concentration at the bottom (Figure 2b). These gradients can be explained by the TEM-sample geometry, where the QD core is roughly sliced in the middle along the [1–10] orientation (Figure S3a). Then, the electron beam detects about half of the [110] orientation of the nanohole in projection. As a result, the Ga signal decreases toward the bottom of the QD. In contrast, the Al signal may stem from (i) the AlAs sidewall, (ii) the Al_0.23_Ga_0.77_As layer, and (iii) possible intermixing of AlAs and GaAs. The latter would result in an Al_*x*_Ga_1–*x*_As layer around the QD core, but this could not be clarified. A visible O signal is present on the film surface due to sample oxidation. The oxidized layer, approximately 5 nm (Figure S6), has an amorphous structure from natural oxidation and likely consists of ∼2 nm of deposited GaAs, followed by a ∼1 nm Al droplet-forming AlAs layer, and a ∼1–2 nm Al_0.23_Ga_0.77_As matrix layer, similar to the structure reported by Toyoshima et al.^53^

Higher-magnification STEM imaging reveals that the QD protrudes approximately 3 nm above the surrounding Al_0.23_Ga_0.77_As layer (Figure 2d,e), consistent with the statistical results (Figure S1f). The absence of intensity differences around the QD in the low-angle annular dark-field (LAADF) STEM images (Figure 2d) suggests the absence of or low strain fields^54,55^ at the QD-Al_0.23_Ga_0.77_As (or AlAs) interface. The GaAs QD grows coherently inside the nanohole in Al_0.23_Ga_0.77_As, and an Al-rich layer is expected in between. In contrast, the cloud-like intensity variations visible in Figure 2d,e are caused by slight thickness variations and possible contamination of the TEM sample. The HAADF-STEM image in Figure 2e shows no intensity variations between the Al_0.23_Ga_0.77_As layer and QD, which contrasts with Figure 2a. The discrepancy between Figure 2a and 2e can be attributed to the higher electron-beam current used during STEM-EDS mapping and for acquiring the HAADF-STEM image in Figure 2a. The higher signal-to-noise ratio in the latter compared to Figure 2e reveals the slight changes in layer composition resulting in different *Z*-contrast. High-resolution HAADF-STEM imaging of the QD-Al_0.23_Ga_0.77_As interface shows no crystalline defects (Figure 2f). Note that the displayed image was denoised.^56^ Together with the LAADF-STEM signal in Figure 2d, which shows no contrast variations indicative of such defects or strain, we conclude that the QD-Al_0.23_Ga_0.77_As interface is highly coherent for the analyzed QD, which provides a stable environment for subsequent selective etching.

To further confirm the 3D morphology features of DENI QDs, we performed a selective chemical etching procedure (Supporting Information). Citric acid and hydrogen peroxide can be used for selective etching of GaAs through an oxidation–reduction reaction, known for its high selectivity to GaAs over AlAs.^40^ However, the selectivity to AlGaAs can reach up to 100 depending on the Al concentration,^35^ with slight modifications to etchant ratios causing substantial changes. It is worth noting that the rate of etching is influenced by material defects, crystal orientation, and surface finish, resulting in the creation of distinct features, such as etch pits or hillocks.^34^ Therefore, the rough surface of elliptical QDs will be more easily etched into “pits”, which will further enhance etching. TEM analysis revealed Al-rich sidewalls which are expected to prevent etching and exhibit a nanohole-like morphology. The etching morphologies are divided into three stages as shown in Figure 3.

**Figure 3:**
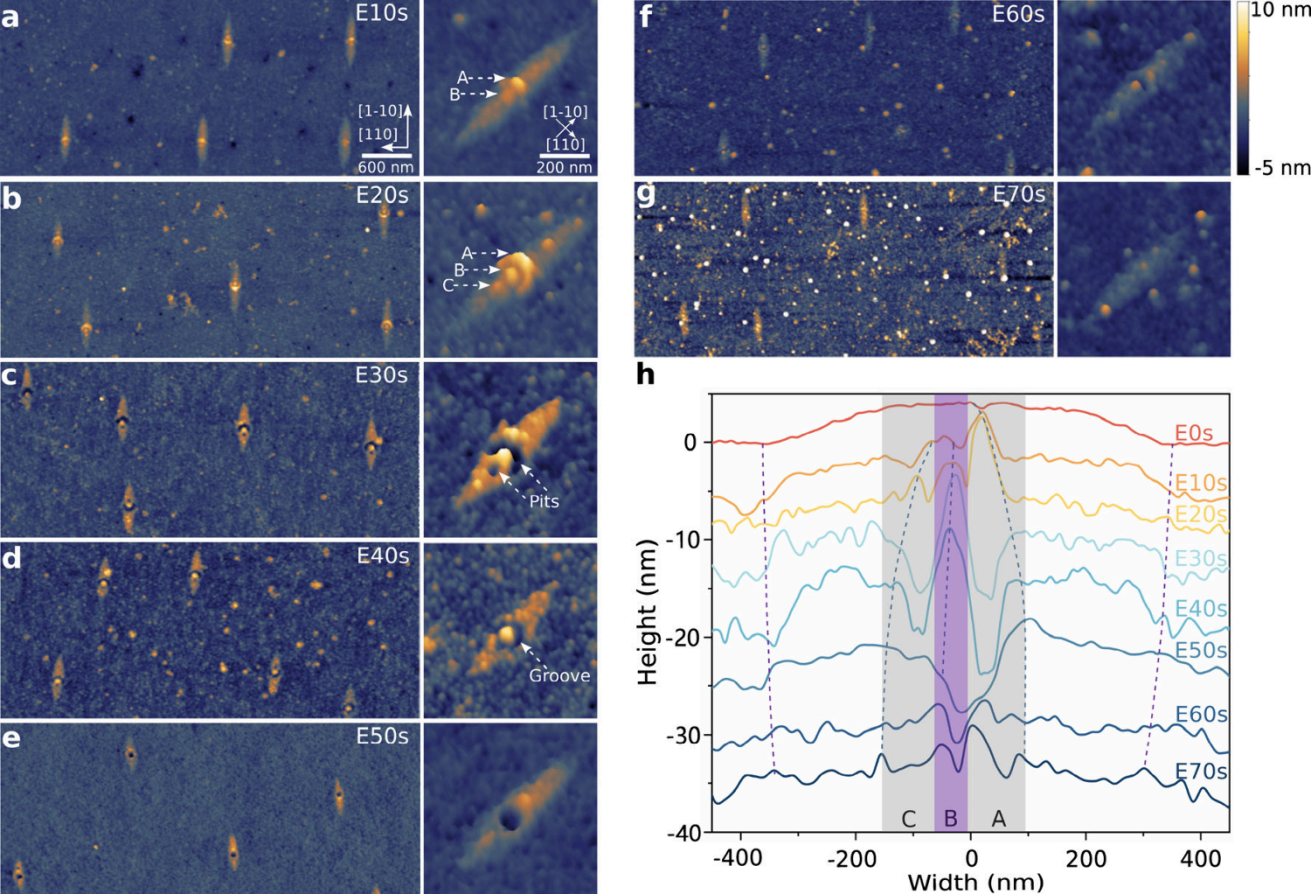
Morphology evolution of the QD by selective chemical etching and AFM. (a) to (g) represent the surface morphology changes after etching 10 to 70 s (E10s to E70s), respectively. The tilted view (30°) of individual QD images on the right is from the top view QDs (a) to (g). (h) Line profiles in [1–10] orientation are based on the morphological changes from (a) to (g), with the center of the elliptical outline as the benchmark and the height change curve under various etching conditions. The width of the elliptical platform in the [1–10] orientation after etching is shown by the purple dashed line, which changes in size from 700 nm (190 nm) to 660 nm (180 nm) in [1–10] ([110]) orientation. The gray dashed lines demonstrate the trends of the A, B, and C peaks which are also marked by gray and purple areas, respectively.

First, Figure 3a shows that the elliptical bump is reduced, revealing distinct, well-defined structures in the middle: a sharp higher bulge (peak-A) and a lower bulge (peak-B) emerging along the [1–10] orientation. After the second etching, peak-A persists and evolves into a crescent-shaped structure surrounding peak-B. A new peak (peak-C) appears next to peak-B, opposite to peak-A (Figure 3b). We believe the surface oxide is rapidly etched away, unrevealing pits in the region between the peaks. Peak-A and peak-C are distinguishable as taller crescent- and shorter separate-structure, respectively. They resemble the nanoring structure of the nanohole but are opposite in height, indicating that the crescent ring grows faster and merges with the separate ring. This also explains the crossover for the main facet angles in the inset of Figure 1f. At a nanohole fabrication temperature of 635 °C, the presence of facets along the inner wall may result in stacking faults and enhance the etching rate.^36,38,39^ The appearance of the hillock peak-B further verifies the existence of multiangle facets on the sidewall near the nanohole opening.

Second, following the third etching cycle, significant erosion occurs in the pit regions between the peaks A and B and between peaks B and C as evidenced by the formation of larger pits (Figure 3c). After the fourth etching, the pits merged, resulting in the formation of an annular “groove” surrounding the central blunt peak-B (Figure 3d). In the fifth etching, peak-B is etched away (Figure 3e), resulting in a crater-like shape from the top view. The line scans from the E20s to the E40s indicate significant erosion and increased lateral dimensions between peak-A and peak-C (dotted gray line in Figure 3h). The well-defined groove observed along the sidewall, with an increasing depth correlated to the number of etching cycles, suggest the possible manifestation of asymmetrical Al(Ga)As distribution. Figure 3h E50s shows that the groove is etched preferentially toward the right, ending at peak-A. This indicates that the left side corresponds to an Al-richer sidewall. The Al-richer sidewalls extend to the surface at peak-C, while peak-B represents filled GaAs, based on comparison with the TEM results. The etching rates of the (111)A and (111)B planes in the zinc-blende structure are nonisotropic due to their termination in different atomic layers along the polar stacking direction.^57^ However, the main facet at the bottom of the nanohole is dominated by a large angle (111),^36^ so the overall etch rate is lower and almost the same in both orientations. But the continuous increase in the lateral dimensions of groove indicates that the sidewall has accelerated etching characteristics, which is attributed to the possible presence of Al_*x*_Ga_1–*x*_As. The disappearance of peak-B is evidence that the bottom sidewall etch rate is lower than that of GaAs.

Third, the surface of the sample becomes rougher after the sixth and seventh etching cycles. Only elliptical platform imprints are present due to the combination of deposited GaAs with surface AlAs, forming Al_*y*_Ga_1–*y*_As with high Al content around the nanoring, and the simultaneous generation of Al_2_O_3_^40^ byproducts, which prevent etching. The ring-shaped wall surrounding the hole opening is not discernible, as shown in Figures 3f and 3g. The protruding parts in the middle of the E60s and E70s curves indicate that the bottom of the inverted cone has not been completely etched away, and based on the Al signal of TEM and EDS, it can be inferred that this bottom of the inverted cone is also an Al-richer region.

Physical structure modeling visualizes the composition and distribution of the AlGaAs alloy, aiding in exploring QD morphology impact on optical properties. Figure 4 presents a true-scale 3D view of the DENI GaAs/AlGaAs QD, consistent with all measured data. The etching process reveals that the majority of nanohole sidewalls are not uniformly thick, with the thicker and Al-richer sidewall extending from the bottom of the inverted cone to the surface nanoring. This indicates that the QD filled in the nanohole is surrounded by several nanometers of Al_*x*_Ga_1–*x*_As (*x* ≫ 0.23) of uneven thickness, within the Al_0.23_Ga_0.77_As barrier. These inhomogeneous AlGaAs alloys introduce possible anisotropic strains that affect the intrinsic asymmetry of the zinc-blende crystal. The asymmetric distribution of Al-rich sidewalls elongates the QDs along the [1–10] orientation, further disrupting the overall structural symmetry.^58^ These two variations lead to more complex exciton degeneracies, which directly cause the splitting of optically allowed (bright) exciton states and increase the FSS.^58^ Second, the quadrupole broadening of arsenic nuclei is sensitive to compositional modulation.^26,59^ The random distribution of Al and Ga atoms at cation sites lead to disorder within the Al_*x*_Ga_1–*x*_As alloy, affecting nuclear spin-flip dynamics; the inhomogeneous strain-induced magnetic field distribution enhances spin–orbit coupling, promoting electron spin flips;^59^ the mixing of GaAs and AlGaAs materials in QDs increases wave function leakage, thereby enhancing interactions with the surrounding matrix;^25,26^ these factors collectively shorten the spin coherence time. Additionally, the random strain alters the band structure, potentially causing shifts in exciton emission wavelengths and resulting in spectral inhomogeneity.^58,60^ Third, the enhanced FSS and increased probability of spin flips lead to more nonradiative recombination pathways, such as possible Auger recombination, which affects the intensity and position of spectral lines.^61^ Experimental evidence suggests that QDs with good optical properties often require smaller size and higher overall symmetry^18,23−25^ and, thus, precise control of DENI growth parameters such as decreasing Al-droplet size, to obtain highly symmetric alloy distribution.

**Figure 4:**
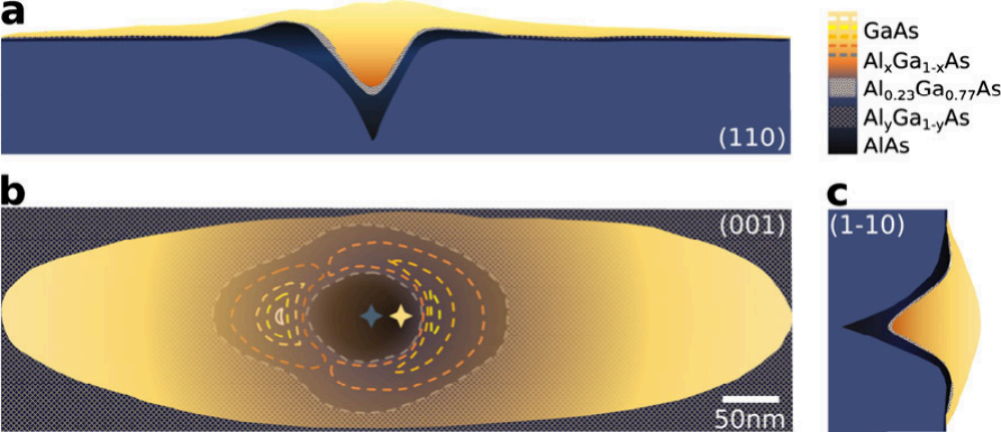
Three views of GaAs/AlGaAs QDs morphology. (a) and (c) are cross-sectional views of the (110) and (1–10) crystal planes, respectively, showing the morphology of nanohole and GaAs QDs. For clarity, the [001] orientation in (a) and (c) is elongated to twice the length of the [110] and [1–10] orientations. (b) is a top-perspective view of the QD. The dark blue area in the center represents the nanohole and nanoring, and the blue star represents the nanohole center. It is worth noting that the center of the formed elliptical bump is biased toward the crescent-shaped part of nanoring and does not coincide with the center of the nanohole. The multiple dashed yellow lines represent the height profile of the nanoring, while the yellow star represents the center of the elliptical bump. The black regular square represents the high Al content Al_*y*_Ga_1–*y*_As, and the dark blue random square area represents the Al_*x*_Ga_1–*x*_As layer with lower but unknown Al concentration.

In this study, we present the first detailed investigation of the morphology of DENI GaAs/AlGaAs QDs. By combining TEM and AFM of chemical etching, we find that the sidewalls of the inverted conical nanoholes are composed of Al-enriched AlGaAs. The Al content is asymmetrically distributed along the nanoholes and in different crystal directions. Additionally, we investigate the crystallinity of the QD/nanohole interface and reveal a uniform crystalline interface. The QD region contains crystalline and homogeneous GaAs. These observations allow for a detailed physical model of the QD morphology, providing a basis for accurately simulating and deterministically optimizing the electronic optical properties of DENI QDs.

## Data Availability

TEM and AFM data used in this study are available on Zenodo. https://zenodo.org/records/11004134

## Supporting Information Available

Sample growth parameters, sample morphology statistics, TEM sample preparation procedures and parameters, elemental and structural analysis of sample layers, and chemical etching procedures (PDF)
