# Supplementary material for: Unveiling the 3D Morphology of Epitaxial GaAs/AlGaAs Quantum Dots
Source: Nano Lett. 2024 Jul 25;24(33):10106–13. doi: 10.1021/acs.nanolett.4c02182 (PMC11342363; doi:10.1021/acs.nanolett.4c02182)
Supplement: Supplementary file 1 — nl4c02182_si_001.pdf [file nl4c02182_si_001.pdf]

Supporting information

Unveiling the 3D Morphology of Epitaxial GaAs/AlGaAs Quantum Dots

Yiteng Zhang[1†], Lukas Grünewald[2†], Xin Cao[1], Doaa Abdelbarey[1], Xian Zheng[1], Eddy Patrick Rugeramigabo[1], Johan Verbeeck[2], Michael Zopf[1,3*], and Fei Ding[1,3]

1. *Institut für Festkörperphysik, Leibniz Universität Hannover, Appelstraße 2, 30167, Hannover, Germany*
2. *EMAT, University of Antwerp, Groenenborgerlaan 171, B-2020 Antwerp, Belgium*
3. *Laboratorium für Nano- und Quantenengineering, Leibniz Universität Hannover, Schneiderberg 39, 30167, Hannover, Germany*

†Authors contributed equally to this work

**Corresponding author: michael.zopf@fkp.uni-hannover.de*

Keywords: GaAs/AlGaAs, semiconductor quantum dots, 3D morphology, HAADF-STEM, selective chemical etching, AFM.

Droplet etching and nanohole infilling

The GaAs/AlGaAs nanostructure studied here is grown on an $Al_{0.23}Ga_{0.77}As$ (001) epi-ready layer, utilizing solid-source molecular beam epitaxy (MBE, Riber Compact 21) with *in-situ* DENI[1] method, as depicted in Figure S 1a. After temporarily interrupting the supply of As_4 for a short period to create an As-poor environment at 635℃, one monolayer Al is deposited and forms droplets on the $Al_{0.23}Ga_{0.77}As$ surface which becomes liquefied beneath the droplets. As diffuses and forms a crystalline wall with Al droplets, surrounding the nanohole due to atomic concentration gradients at the $Al_{0.23}Ga_{0.77}As$ surface and within the Al droplets[2]. The reintroduction of As_4 ensures the complete development of nanoholes on the surface[2]. GaAs migrate into these nanoholes after resuming the simultaneous supply of Ga and As_4, ultimately forming QDs. Finally, the growth temperature is maintained at 625℃ in an As-rich environment for 2 minutes to obtain a smooth surface and prevent over-oxidation of the sample after being removed from the MBE, as shown in Figure S 1b.

The nanohole line profile image in Figure S 1c, captured following the deposition of an Al droplet on an $Al_{0.23}Ga_{0.77}As$ surface at 635 °C, illustrates the formation of the asymmetrical quantum ring surrounding the hole opening, which is predominantly composed of As of the droplet material[3] and is recognized as the essential process for As removal from the substrate. Here, our nanostructures show an asymmetric shape, the heights R of the quantum rings are 2.95 nm and 3.69 nm on average, respectively; the average depth D of the nanohole is 22.2 nm, which remains highly consistent in depth in the 2 directions; however, the nanohole opening dimensions O are slightly different, with an average of 68.4 nm in the [110] orientation and 83.2 nm in the [1-10] orientation, forming an elliptical hole that has a long-to-short ratio of approximately 1.2. For the elliptical quantum dots filled with 1.99 nm, the dimensions and statistics are shown in Figure S 1d and 1f, with an average long axis W1 of 695.6 nm, short axis W2 of 185.5 nm, and a height H of 3 nm. Note that the profile in the extended [1-10] orientation shows an uneven top profile, which is prevalent. All data were taken from the center of the sample and exported by Gwyddion (V2.60).

Previous studies indicate that the asymmetric quantum ring is influenced by factors such as the temperature of the substrate during the DENI etching process, the volume[4,5], deposition rate[6] and composition[7] of the Al droplets, the annealing time after etching[5,7], and As background pressure[7,9]. It can be simply understood that the deeper the hole, the more asymmetric the quantum ring/hole is. We attribute the local asymmetry in our quantum ring to the volume of supplied Al droplets and relatively high etching temperature. High temperatures tend to increase the etching depth of nanoholes, leaving a more asymmetric quantum ring shape at the nanohole opening[6]. For the inner wall of the nanohole, analysis shows that on the one hand, the volume of material preserved within a wall closely corresponds to the material extracted from a hole, collectively constituting approximately 3% of the initial droplet volume[8] . On the other hand, these nanoholes exhibited faceted inner walls covered by a thin layer of $Al_xGa_{1-x}As$, with a thickness of several nanometers[10]. Notably, the composition of this covering layer differs not only from the underlying substrate but also from the quantum ring[10]. In general, it is a relatively mature method to create QDs by etching holes with In, Ga, and Al droplets, and people have already extended it to the InP-based[11] system and AlGaSb-based[12] system.

Finally, a top layer of $Al_{0.23}Ga_{0.77}As$ is grown based on different filling amounts, followed by a GaAs cap layer, and then the QDs spectrum is measured as shown in the Figure S 1g. The exciton emission wavelength of QDs is obviously directly proportional to the filling amount. The full growth parameters of the samples can be seen in Table S 1.

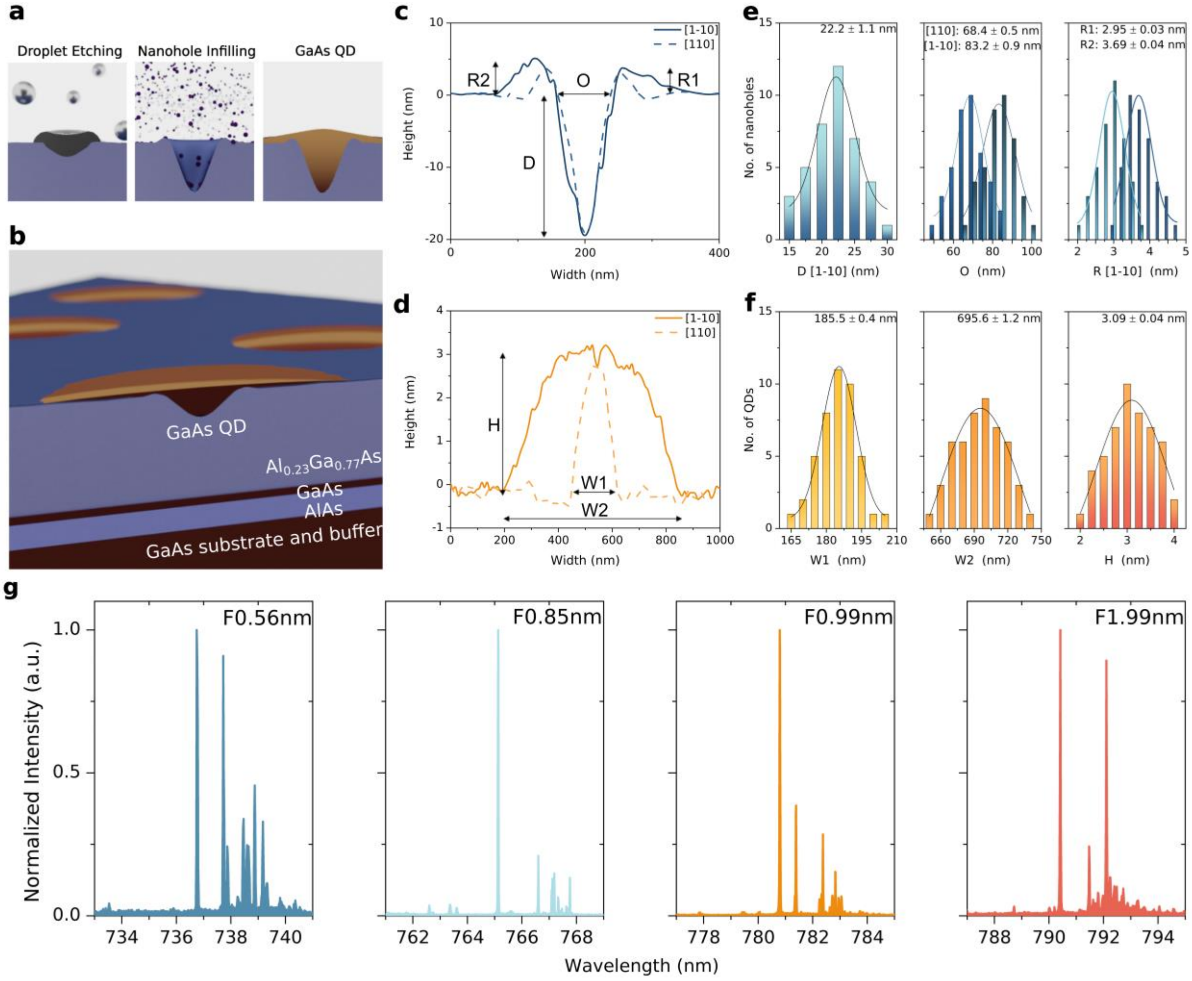

Figure S 1. Statistical comparison of nanohole and quantum dot samples. (a) Schematic of *in-situ* Al droplet etching and nanohole infilling performed on an AlGaAs epi-ready surface. (b) Schematic of the sample cross-section (not to scale). (c) The size of the nanohole and (d) QD after filling 1.99 nm GaAs. (e) and (f) correspond to the size statistical results of nanohole and QD morphology, respectively. Average statistical results are obtained through Gaussian fitting. (g) shows the QDs spectrum under different filling amounts at 4K.

Table S 1. Sample design with relevant MBE growth parameters.

Step No.	Material	Thickness (nm)	Duration (s)	Substrate temperature (°C)	Growth Pressure (torr)	Comments
**1**	GaAs	100	520	575	1.44×10^{-7}	Buffer layer
**2**		200	1040	to 625		
**3**	As	/	10	635		Growth interruption
**4**	AlAs	50	230		to 1.67×10^{-7}	Sacrificial layer
**5**	As	/	10		1.67×10^{-7}	Growth interruption
**6**	GaAs	5	25.8	to 625		Protection layer
**7**	As	/	10	625		Growth interruption
**8**	$Al_{0.23}Ga_{0.77}As$	139	551.4			Barrier layer
**9**		84	333.3			
**10**		15.1	60	to 635		
**11**	As	/	10	635		Growth interruption
**12**	/	/	15		to 3.2×10^{-8}	As close
**13**	Al	0.11	1			Al droplets deposition
**14**	/	/	45			Droplet etching
**15**	As	/	60		to 1.44×10^{-7}	Nanohole crystallization
**16**	GaAs	0.14	4×4		1.44×10^{-7}	Nanohole infilling
**17**	As	/	120	to 625		Growth interruption

TEM characterization of GaAs/AlGaAs QDs

The samples were first coated with 25 nm Carbon in a Leica ACE 600 coater to protect the sample's surface during subsequent sample preparation. Cross-section samples for TEM were prepared by an in-situ lift-out technique[14] in a combined Ga^+ focused ion beam (FIB)/SEM instrument (FEI Helios NanoLab 650). The bulk samples were first aligned along the GaAs substrate major edge and the TEM lamella was lifted out so that the electron beam in the TEM was parallel to the GaAs [110] orientation, i.e., parallel to the short axis of the elliptical bumps. The latter were located on the surface by secondary-electron SEM imaging. The electron- and ion-beam-induced depositions of Pt/C protection layers were used to protect the region of interest from further ion milling. Ion energies ranging from 30 keV down to 2 keV were used for coarse and fine milling, respectively, with a final surface cleaning at 2 keV to minimize the thickness of amorphous surface layers[15]. The sample was carefully thinned to preserve the center of the QD region. More details on TEM sample preparation are given in the supplementary information (Figure S 2).

After preparation, the TEM sample was stored in an Ar glovebox to minimize oxidation, especially oxidation of AlAs[16]. In total, the sample was exposed to air for about 20 min during transfer between microscopes and the glovebox. An FEI Tecnai Osiris equipped with ChemiSTEM technology[17] and operated at an accelerating voltage of 200 kV was used for scanning TEM (STEM) imaging and chemical analysis with energy-dispersive x-ray spectroscopy (EDS). A convergence semi-angle of 10.5 mrad was applied. High- and low-angle annular dark-field (LAADF/HAADF) STEM images were acquired using a Fischione Model 3000 ADF detector with collection-angle ranges of about 65–200 mrad and 14–84 mrad, respectively. The HAADF-STEM intensity roughly scales with the average atomic number $Z^{1.7}$ for constant sample thickness, which leads to directly interpretable Z-contrast images[18,19]. The

LAADF-STEM imaging mode is sensitive to strain fields and crystalline defects[20,21]. The STEM-EDS datasets were collected and analyzed using the Bruker ESPRIT software version 1.9. The shown elemental maps represent the background-corrected net intensities of the x-ray peaks at each scan position. Standard less quantification of EDS spectra was performed within the thin-film approximation using the Cliff-Lorimer approach[22] without correction for x-ray absorption due to unknown or varying sample thickness. The latter aspect will lead to an underestimation of Al due to the absorption of low-energy Al Kα x-rays (1.49 keV) in the sample. In contrast, the Ga concentration will be overestimated due to Ga implantation resulting from Ga^+ FIB preparation.

TEM-Sample Preparation

Figure S 2presents details about the TEM sample preparation of the quantum dots (QDs). Even though a focused ion beam (FIB) instrument enables site-specific sample preparation, preserving the central region of a QD (denoted as “QD core”) is still challenging due to its small lateral dimensions of a few tens of nm. The QDs protrude from the substrate surface and show an elliptical shape (Figure S 2a, marked with arrowheads). The QDs can be located in conventional secondary-electron scanning electron microscopy (SE-SEM) imaging, here at 5 keV electron energy, and imaged in the field-free mode of the microscope. The round shapes visible in Figure S 2a are caused by a partial detachment of the deposited 25 nm thin C film (using a Leica ACE600 coater) under electron beam irradiation (see also the scheme in Figure S 2b). A QD without detached C film was selected for TEM sample preparation, e.g., as shown in Figure S 2c. Scanning the electron beam just over the QD region for a few ten seconds generates an additional C layer due to sample contamination (Figure S 2d). This marks the QD size and alignment during further FIB preparation. The FIB preparation aims to thin the TEM sample (targeting about 75 nm thickness, Figure S 2d) so that the QD core is preserved. Note that the QD core could not be imaged directly with SEM during FIB preparation due to its small size (extending about 20 nm into the substrate) and its similar chemical composition (GaAs QD vs. $Al_{0.23}Ga_{0.77}As$ substrate) so that verification of the sample integrity had to be done using TEM. The schematic diagram of the TEM sample after preparation is shown in Figure S 3.

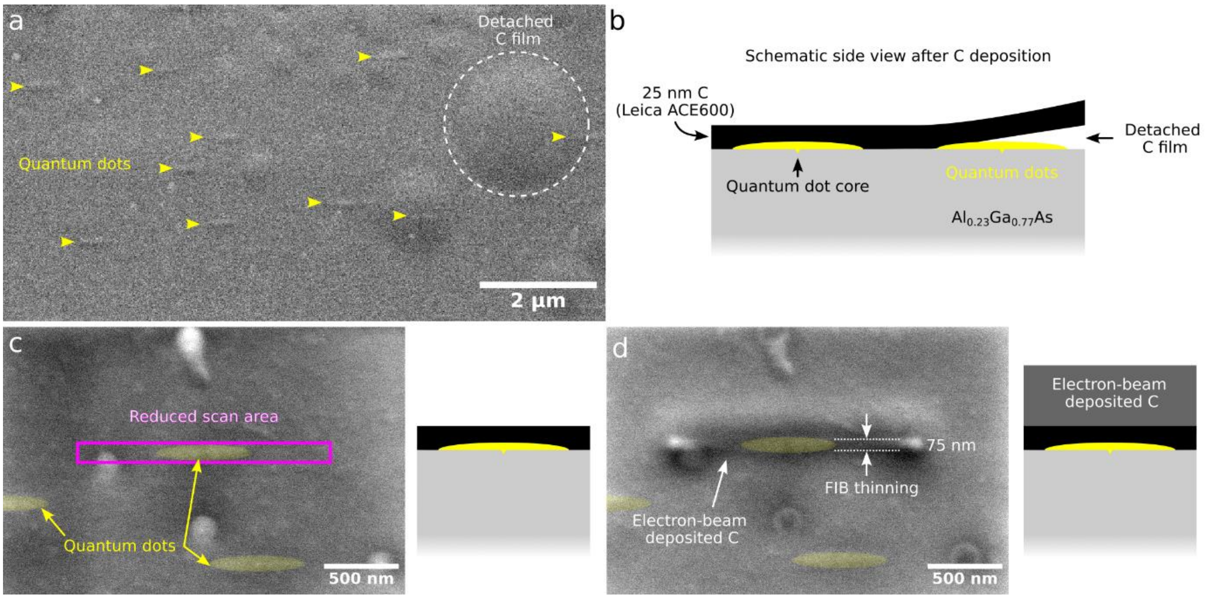

Figure S 2. Preparation of TEM samples with a combined FIB/SEM instrument. (a) Overview secondary electron (SE) SEM image (5 keV) of the C-coated surface. The QDs have an elliptical shape (arrowheads). Bubbles form under electron-beam irradiation due to detaching of the deposited C film (marked with a dashed circle). (b) Schematic cross-section of the situation in (a) with partially detaching C film. The core of the quantum dot is located roughly in the center. (c) Higher magnification SE-SEM image of a selected QD. Continuous scanning in an area of the QD will lead to C-rich contamination which acts as a marker during final FIB thinning. (d) Same QD as in (c) after SEM scanning and deposition of an additional C film. The dotted lines roughly show the target thickness of the targeted TEM-sample thickness of about 75 nm.

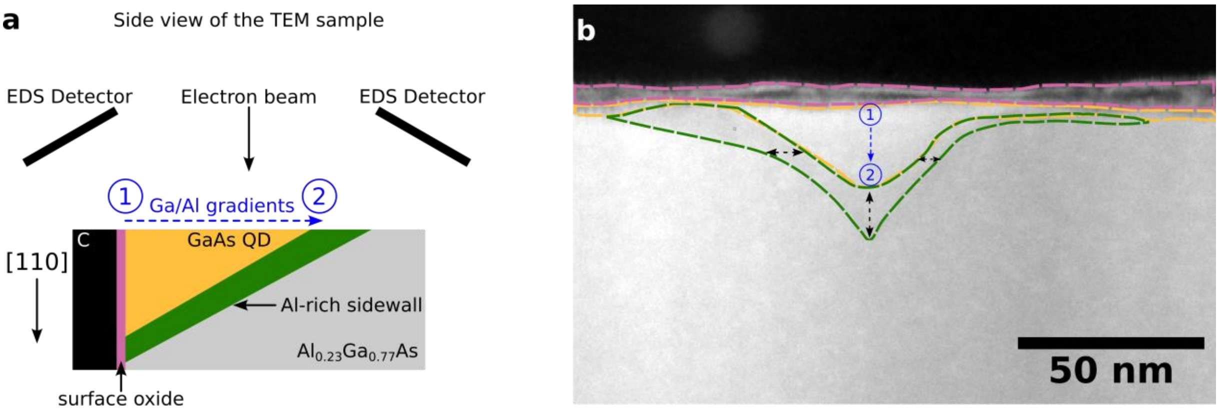

Figure S 3. Schematic of TEM-sample geometry. (a) Side-view schematic of possible TEM-sample geometry in the TEM. The electron beam direction is top to bottom. The EDS detectors are placed above the TEM sample and are only schematically shown here (not to scale). The GaAs QD may be sliced roughly halfway along the [110] direction after TEM-sample preparation. The measured EDS and STEM signals arise from the whole sample along the electron beam direction. At region (1), a high Ga and low Al concentration is measured, and vice versa at region (2). This results in the observed concentration gradients in Ga and Al maps in (b). Al signal stems from $Al_{0.23}Ga_{0.77}As$, the AlAs sidewall, and possibly an $Al_xGa_{1-x}As$ intermixing at the QD wall (not shown here). Note that low-energy Al Kα (1.49 keV) may be partly absorbed in the TEM sample on the way to the EDS detector, especially when originating closer to the bottom of the sample (e.g., from AlAs and $Al_{0.23}Ga_{0.77}As$ in region (1)). (b) TEM sample cross-section HAADF image in Figure 2a in the main text. Dotted lines assist in observing the outline of each part. The dashed arrows indicate non-uniformity in sidewall thickness.

Layer Composition and Microstructure

The composition of the epitaxial grown layers – AlAs/GaAs/$Al_{0.23}Ga_{0.77}As$ grown on a GaAs substrate, below the QDs were analyzed with STEM-EDS (Figure S 4). The expected elemental distributions are visible at medium and high magnifications (Figure S 4a and 4b, respectively). Notably, the thin GaAs layer (about 4 nm thickness) on top of AlAs is revealed by an Al depletion. The quantified chemical composition of the layers is within expectation and the error range of standard less EDS quantification (Table S 2). Summed-up EDS spectra from the respective layers shown in Figure S 4a were used for quantification to maximize the signal-to-noise ratio in the EDS spectra.

The sample surface is oxidized, resulting in a pronounced O signal. Further below, the surface layer is discussed in more detail (Figure S 6). Otherwise, the O signal is uniform in the sample and stems from possible contamination and slight oxidation of the TEM sample during transfer between microscopes, and a small part of oxidation may come from the background oxidation during the nanohole crystallization process in the MBE chamber. It is noteworthy that the AlAs was oxidizing faster than the other layers when the TEM sample was reinvestigated after storage in air (not shown here).

The epitaxial growth of the layers is confirmed by selected-area electron diffraction (SAED, Figure S 5a and 5b) and HAADF-STEM imaging (Figure S 5c). In SAED, the diffraction spots of all three epitaxial grown layers (AlAs/GaAs/$Al_{0.23}Ga_{0.77}As$) overlap with the underlying GaAs substrate due to the small change in lattice parameter and the limited angular resolution of the shown SAED pattern (Figure S 5b). The acquisition region of the latter is determined by the SAED aperture shown in Figure S 5a, containing all layers and the substrate. The (denoised[23]) HAADF-STEM image shows the atomic arrangement at the interfaces between the layers, and the resolved atomic columns confirm the crystallinity of the layers.

The elemental distribution across the oxidized sample surface consists of multiple layers (Figure S 5a and 5b). At the top, the C protection layer from TEM-sample preparation is visible. Then, the actual sample surface starts with a Ga- and O-rich layer without As, which is denoted as (I) in Figure S 6b. This indicates the formation of layer Ga_xO_y. Below the latter, an As-rich and Ga-depleted layer is observed, cf. (II) in Figure S 6b. Al is detected in both layers, (I) and (II), and decreases gradually away from $Al_{0.23}Ga_{0.77}As$. The total width of layers (I) and (II) is about 5 nm. The oxidation of $Al_{0.23}Ga_{0.77}As$ might be similar to that reported for GaAs, which forms (I) Ga_2O_3 and (II) elemental As[24,25]. The shown results are in good agreement with the STEM-EDS measurements by Toyoshima et al.[26]

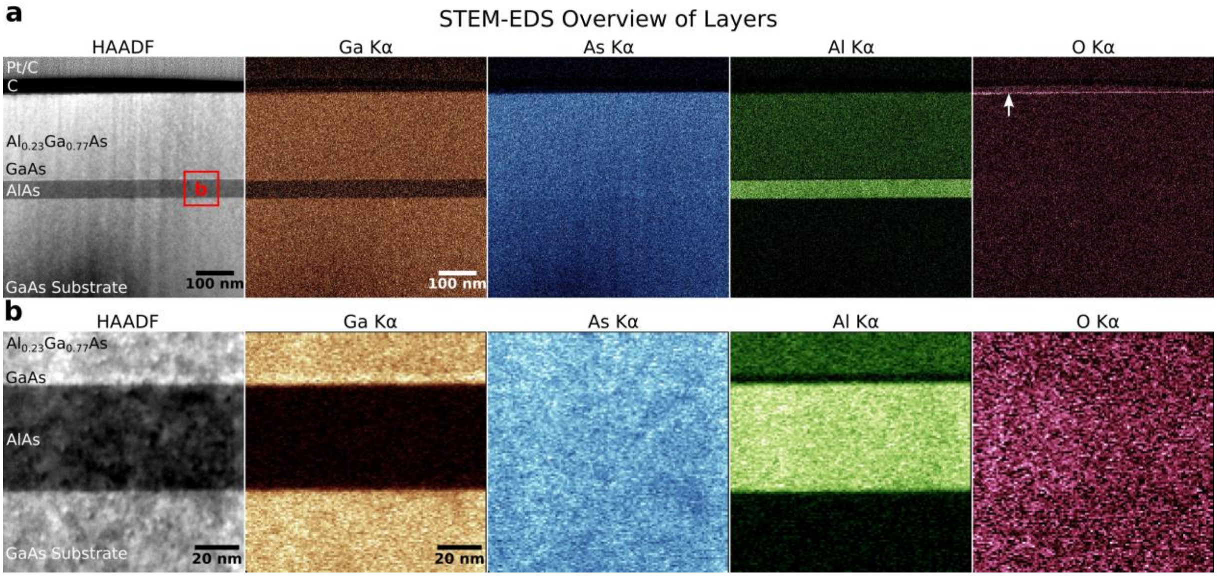

Figure S 4. Elemental maps of the epitaxial grown layers at (a) medium and (b) higher magnification obtained by STEM-EDS. Overall, the expected elemental distribution is observed. The sample surface is oxidized, resulting in a pronounced O signal at the surface (marked by an arrow in the O map in (a)). The O signal in other sample regions is uniform and stems from sample contamination and slight oxidation of the TEM sample during transfer between different microscopes.

Table S 2. Quantified chemical composition from STEM-EDS with nominal values shown in brackets. Standard less quantification with normalization to 100 at% was used without x-ray absorption correction. The latter leads to an underestimation of Al. In contrast, TEM-sample preparation by a Ga^{+}-FIB leads to Ga implantation resulting in Ga overestimation.

Region	Ga / at%	As / at%	Al / at%
**$Al_{0.23}Ga_{0.77}As$**	39.4 (38.5)	49.9 (50.0)	10.7 (11.5)
**AlAs**	1.9 (0)	50.7 (50.0)	47.4 (50.0)
**GaAs Substrate**	51.4 (50.0)	48.3 (50.0)	0.3 (0)

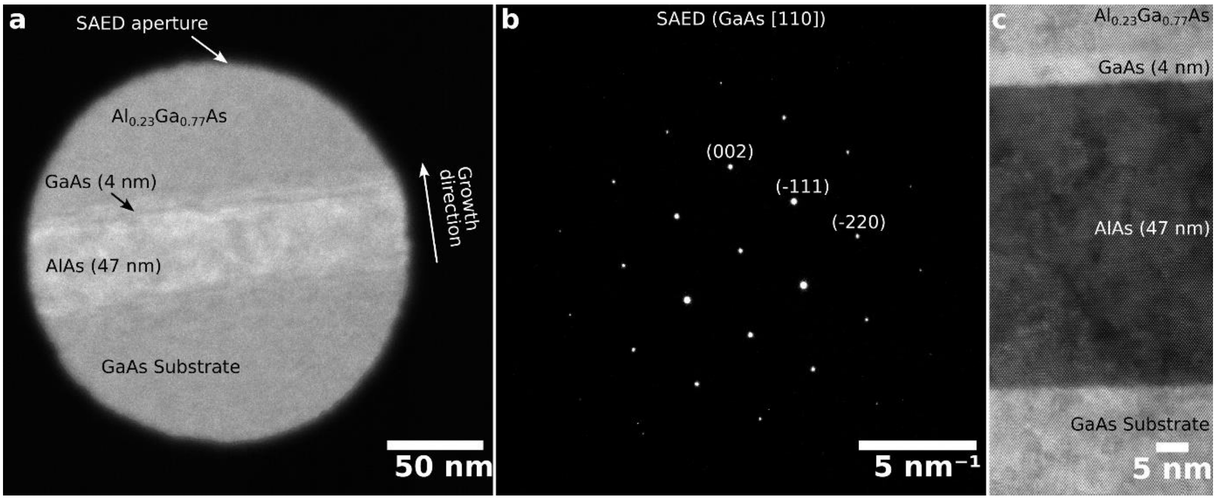

Figure S 5. Microstructural TEM analysis of buried sample layers. (a) TEM bright-field image with inserted selected-area electron diffraction (SAED) aperture showing the signal area of different material layers contributing to the SAED pattern in (b). (b) SAED pattern from the region in (a) showing only reflections from GaAs<110>-type zone-axes orientation. No additional spots are visible due to the similar lattice constant for all layers in (a), essentially overlapping in the shown SAED pattern. The absence of other spots confirms epitaxial

growth. (c) High-resolution (denoised[23]) HAADF-STEM image of the layers shown in (a) confirming the crystallinity and epitaxial growth of the layers on the GaAs substrate.

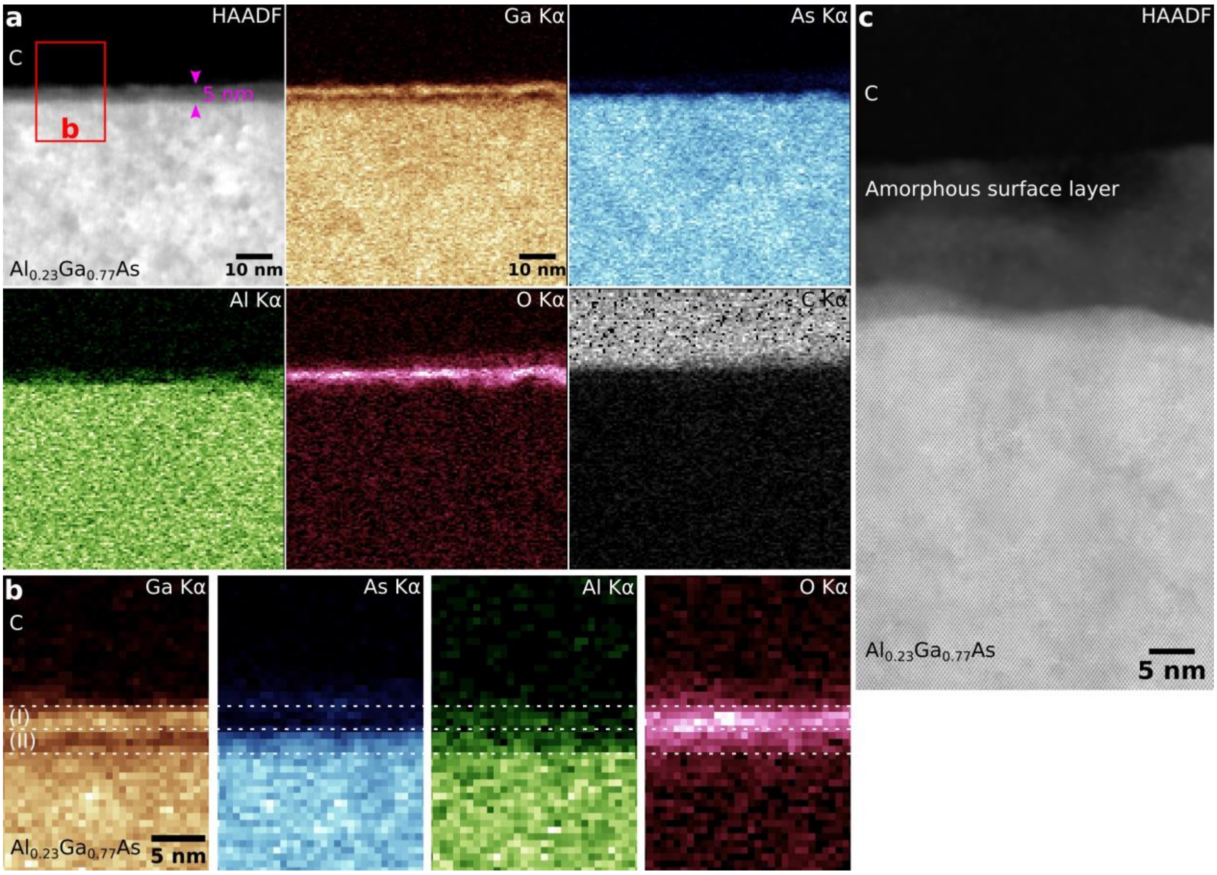

Figure S 6. Chemical analysis of the oxidized surface layer of $Al_{0.23}Ga_{0.77}As$. (a) and (b) Medium and high magnification STEM-EDS elemental maps showing an (I) Ga/O-rich top layer followed by a (II) Ga-depleted, As-containing layer. The Al signal decreases gradually away from $Al_{0.23}Ga_{0.77}As$ towards the surface. (c) HAADF-STEM image showing the crystalline $Al_{0.23}Ga_{0.77}As$ layer at the bottom, the amorphous surface layer, and the C protection layer.

Selective chemical etching

Standard procedures for precise control of depth etching are key to repeatable and controllable selective etching. Initially, a gold marker was deposited on the sample surface to demarcate the etching region. Each marked area, approximately 2 mm^2 in size, ensured a comprehensive reaction between the etching solution and the sample surface. Subsequently, the entire sample surface was coated with a 1 µm thick photoresist, selectively exposing only region 1 through photolithography for subsequent etching. The wet chemical etching solution comprised 12.5g citric acid ($C_6H_8O_7$, 99.5%) and 1 ml hydrogen peroxide (H_2O_2, 31%), diluted with 37.5 g of water. This selective etchant enables GaAs to be etched at a higher etching rate than Al(Ga)As alloys[27]. The solution was prepared at room temperature and the components were mixed for 15 minutes under mechanical stirring. The sample was immersed in the solution for 10 seconds, followed by an immediate rinse with water to stop further etching. Subsequently, region 2 of the sample was exposed and immersed in the solution so that region 1 and region 2 were etched simultaneously. These steps were iteratively performed to capture the entire morphological evolution of the QDs until their disappearance. We etched seven regions on the elliptical bump

samples, where region 1 was etched seven times, and subsequent regions were etched sequentially with one less time, resulting in a staircase-like structure in the final sample. The etched areas were then named according to the etching time, with region 1 being E70S, region 2 being E60S, and so on. Surface roughness measurements in tapping mode were sequentially conducted in each region to generate a comprehensive morphology change map.

Supporting information references

1. Keil, R.; Zopf, M.; Chen, Y.; Bianca, H.; Jiaxiang, Z.; Fei, D.; Oliver, G. S. Solid-state ensemble of highly entangled photon sources at rubidium atomic transitions. *Nature communications* **2017**, *8* (1): 15501, DOI: 10.1038/ncomms15501.
2. Gurioli, M.; Wang, Z.; Rastelli, A.; Kuroda, T.; Sanguinetti, S. Droplet Epitaxy of Semiconductor Nanostructures for Quantum Photonic Devices. *Nature materials* **2019**, *18* (8), 799–810, DOI: 10.1038/s41563-019-0355-y.
3. Heyn, C.; Bartsch, T.; Sanguinetti, S.; Jesson, D.; Hansen, W. Dynamics of Mass Transport during Nanohole Drilling by Local Droplet Etching. *Nanoscale Res Lett* **2015**, *10* (1), 67, DOI: 10.1186/s11671-015-0779-5.
4. Küster, A.; Heyn, C.; Ungeheuer, A.; Juska, G.; Tommaso Moroni, S.; Pelucchi, E.; Hansen, W. Droplet Etching of Deep Nanoholes for Filling with Self-Aligned Complex Quantum Structures. *Nanoscale Res Lett* **2016**, *11* (1), 282, DOI: 10.1186/s11671-016-1495-5.
5. Heyn, C.; Schnüll, S.; Hansen, W. Scaling of the Structural Characteristics of Nanoholes Created by Local Droplet Etching. *Journal of Applied Physics* **2014**, *115* (2) , DOI: 10.1063/1.4861722.
6. Küster, A.; Heyn, C.; Ungeheuer, A.; Juska, G.; Moroni, S. T.; Pelucchi, E.; Hansen, W. Droplet Etching of Deep Nanoholes for Filling with Self-Aligned Complex Quantum Structures. *Nanoscale Res Lett* **2016**, *11* (1), 282, DOI: 10.1186/s11671-016-1495-5.
7. Fuster, D.; González, Y.; González, L. Fundamental Role of Arsenic Flux in Nanohole Formation by Ga Droplet Etching on GaAs (001). *Nanoscale Res Lett* **2014**, *9* (1), 309, DOI: 10.1186/1556-276X-9-309.
8. Shen, J.; Lv, H.; Ni, H.; Liu, H.; Su, X.; Zhang, J.; Shang, X.; Zhuo, Z.; Li, S.; Chen, Y.; Sun, B.; Zhang, Y.; Niu, Z. Study on the Asymmetry of Nanopore in Al Droplet Etching. *Opt Quant Electron* **2021**, *53* (8), 412, DOI: 10.1007/s11082-021-03011-w.
9. Heyn, C.; Zocher, M.; Schnüll, S.; Hansen, W. Role of Arsenic During Aluminum Droplet Etching of Nanoholes in AlGaAs. *Nanoscale Res Lett* **2016**, *11* (1), 428, DOI: 10.1186/s11671-016-1648-6.
10. Vonk, V.; Slobodskyy, T.; Keller, T. F.; Richard, M.-I.; Fernández, S.; Schulli, T.; Heyn, C.; Hansen, W.; Stierle, A. Faceting of Local Droplet-Etched Nanoholes in AlGaAs. *Phys. Rev. Materials* **2018**, *2* (10), 106001, DOI: 10.1103/PhysRevMaterials.2.106001.
11. Cao, X.; Zhang, Y.; Ma, C.; Wang, Y.; Brechtken, B.; Haug, R. J.; Rugeramigabo, E. P.; Zopf, M.; Ding, F. Local Droplet Etching on InAlAs/InP Surfaces with InAl Droplets. *AIP Advances* **2022**, *12* (5) , DOI: 10.1063/5.0088012.
12. Hilska, J.; Chellu, A.; Hakkarainen, T. Nanohole Etching in AlGaSb with Gallium Droplets. *Crystal Growth & Design* **2021**, *21* (4), 1917–1923, DOI: 10.1021/acs.cgd.1c00113.
13. Leroux, F.; Evoagil, R.; Verbeeck, J. Each Atom Counts: Protect Your Samples Prior to FIB Processing. https://www.leica-microsystems.com/science-lab/life-science/each-atom-counts-protect-your-samples-prior-to-fib-processing/ (accessed 2023-06-16).
14. Langford, R. M.; Clinton, C. In Situ Lift-out Using a FIB-SEM System. *Micron* **2004**, *35* (7), 607–611, DOI: 10.1016/j.micron.2004.03.002.
15. Mayer, J.; Giannuzzi, L. A.; Kamino, T.; Michael, J. TEM Sample Preparation and FIB-Induced Damage. *MRS bulletin* **2007**, *32* (5), 400–407, DOI: 10.1557/mrs2007.63.
16. Taylor, J. A. An XPS Study of the Oxidation of AlAs Thin Films Grown by MBE. *Journal of Vacuum Science and Technology* **1982**, *20* (3), 751–755, DOI: 10.1116/1.571450.
17. Schlossmacher, P.; Klenov, D. O.; Freitag, B.; Von Harrach, H. S. Enhanced Detection Sensitivity with a New Windowless XEDS System for AEM Based on Silicon Drift Detector Technology. *Microscopy today* **2010**, *18* (4), 14–20, DOI: 10.1017/S1551929510000404.
18. Pennycook, S. J. Z-Contrast Stem for Materials Science. *Ultramicroscopy* **1989**, *30* (1–2): 58–69, DOI: 10.1016/0304-3991(89)90173-3.

19. Muller, D. A. Structure and Bonding at the Atomic Scale by Scanning Transmission Electron Microscopy. *Nature materials* **2009**, *8* (4), 263–270, DOI: 10.1038/nmat2380.
20. Yu, Z.; Muller, D. A.; Silcox, J. Study of Strain Fields at A-Si/c-Si Interface. *Journal of Applied Physics* **2004**, *95* (7), 3362–3371, DOI: 10.1063/1.1649463.
21. Fitting, L.; Thiel, S.; Schmehl, A.; Mannhart, J.; Muller, D. A. Subtleties in ADF Imaging and Spatially Resolved EELS: A Case Study of Low-Angle Twist Boundaries in $SrTiO_3$. *Ultramicroscopy* **2006**, *106* (11–12), 1053–1061, DOI: 10.1016/j.ultramic.2006.04.019.
22. Cliff, G.; Lorimer, G. W. The Quantitative Analysis of Thin Specimens. *Journal of Microscopy* **1975**, *103* (2), 203–207, DOI: 10.1111/j.1365-2818.1975.tb03895.x.
23. Lobato, I.; Friedrich, T.; Van Aert, S. Deep Convolutional Neural Networks to Restore Single-Shot Electron Microscopy Images. *npj Computational Materials* **2024**, *10* (1), 10, DOI: 10.1038/s41524-023-01188-0.
24. Mizokawa, Y.; Komoda, O.; Miyase, S.; Long-time air oxidation and oxide-substrate reactions on GaSb, GaAs and GaP at room temperature studied by X-ray photoelectron spectroscopy. *Thin Solid Films* **1988,** *156* (1): 127-143, DOI: 10.1016/0040-6090(88)90288-X.
25. Thurmond, C. D.; Schwartz, G. P.; Kammlott, G. W.; Schwartz, B. GaAs oxidation and the Ga-As-O equilibrium phase diagram. *Journal of the Electrochemical Society* **1980**, *127* (6): 1366, DOI: 10.1149/1.2129900.
26. Toyoshima, R.; Murakami, S.; Eguchi, S.; Amemiya, K.; Mase,K.; Kondoh, H. Initial oxidation of GaAs (100) under near-realistic environments revealed by in situ AP-XPS. *Chemical Communications* **2020**, *56* (94): 14905-14908, DOI: 10.1039/d0cc05279e.
27. Clawson, A. R. Guide to references on III–V semiconductor chemical etching. *Materials Science and Engineering: R: Reports* **2001**, *31* (1-6): 1-438, DOI: 10.1016/S0927-796X(00)00027-9.
